# The Influence of Lipopolysaccharide O‐Antigen Chain Length on Biofilm Formation Capacity and Outer Membrane Proteome Shape of *Salmonella* Enteritidis

**DOI:** 10.1111/1758-2229.70211

**Published:** 2025-11-20

**Authors:** Eva Krzyżewska‐Dudek, Bartłomiej Dudek, Katarzyna Kapczyńska, Paweł Pasikowski, Malwina Brożyna, Justyna Paleczny, Agata Mikołajczyk‐Martinez, Adam Junka, Jacek Rybka

**Affiliations:** ^1^ Department of Immunology of Infectious Diseases Hirszfeld Institute of Immunology and Experimental Therapy, Polish Academy of Sciences Wrocław Poland; ^2^ Platform for Unique Model Applications (PUMA), Division of Translational Technologies, Faculty of Pharmacy Wroclaw Medical University Wrocław Poland; ^3^ Captor Therapeutics Wrocław Poland; ^4^ Department of Biochemistry and Molecular Biology, Faculty of Veterinary Medicine Wroclaw University of Environmental and Life Sciences Wrocław Poland

**Keywords:** biofilm, outer membrane proteome, O‐antigen chain length, *Salmonella* Enteritidis

## Abstract

Biofilm formation is a phenomenon of great medical importance, also affecting food production. In the present work, we investigated the effect of the O‐antigen length of lipopolysaccharide (LPS) of *Salmonella* Enteritidis on biofilm production and the physicochemical properties of *Salmonella* cells, using bacterial deletion mutants. We also analysed the influence of LPS O‐antigen shortening on the composition of the outer membrane (OM) proteome of *S.* Enteritidis. We have shown that the shortening of the LPS O‐antigen part is associated with decreased biofilm biomass formation in some mutants and that it also depends on the composition of the culture medium. Physicochemical properties of bacterial cells changed with the shortening of the O‐antigen, promoting bacterial aggregation and influencing their hydrodynamic size, zeta potential, or hydrophobicity. We have also shown that shorter O‐antigen alters the bacterial proteome in comparison to regular size O‐antigen: flagellar FliC protein was down‐regulated in most mutants, while the HptG as well as 50S ribosomal protein L7/L12 protein were up‐regulated, suggesting increased protein synthesis activity. In some mutants, proteins involved in LPS biosynthesis were also upregulated: lipopolysaccharide core heptose(II)‐phosphate phosphatase, acyl carrier protein, and undecaprenyl‐phosphate alpha‐N‐acetylglucosaminyl 1‐phosphate transferase, implying that the increased LPS biosynthesis is aimed at the replacement of the lacking LPS modal fractions in the *S.* Enteritidis mutants.

## Graphical abstract

Shortening of the LPS O‐antigen part is related to decreased biofilm biomass formation, which also depends on the composition of the culture medium. Physicochemical properties of bacterial cells changed with the shortening of the O‐antigen, promoting bacterial aggregation, hydrodynamic size, zeta potential, and hydrophobicity. Additionally, shorter O‐antigen alters the bacterial proteome in comparison to regular‐sized O‐antigen.

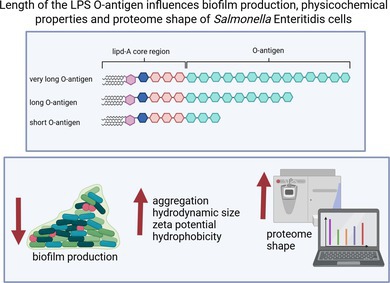

## 1 Introduction

In year 2021, the reported incidence of salmonellosis (disease caused by *Salmonella* sp.) in the European Union was 16.6 cases per 100,000 population, ranking this pathological entity as the second most common gastrointestinal infection (ECDC 2022). The European Food Safety Authority (EFSA) estimates that economic costs related to salmonellosis consume more than 3 billion euros per year (EFSA 2023). This highlights salmonellosis as a disease of significant concern due to both its prevalence and the substantial economic burden it imposes, underscoring the urgent need for enhanced preventive and control measures.

*Salmonella* Enteritidis (*S*. Enteritidis) is one of the most prevalent etiological factors of salmonellosis (European Food Safety Authority and European Centre for Disease Prevention and Control 2021). In humans, infection caused by these bacteria usually occurs via the faecal‐oral route. The sources of infection are contaminated animal products, in particular eggs, pork, and poultry meat. The course of salmonellosis in poultry is often asymptomatic, which increases the risk of unnoticed *Salmonella* spread (Janssens et al. 2008; Hood and Zottola 1997; Joseph et al. 2001; Lee Wong 1998). On the contrary, in humans, salmonellosis manifests in a variety of clinical forms such as gastroenteritis, enteric fever, extra‐intestinal complications, or even bacteriemia and sepsis (Darby and Sheorey 2008; Teklemariam et al. 2023).

On both biotic and abiotic surfaces, the growth of *Salmonella* is associated with its ability to form a biofilm. This refers to a community of bacterial cells that adhere to a surface and form an extracellular, polymeric matrix that protects them from the immune system, antibiotics, or antiseptic and disinfection agents. During the early stages of *Salmonella* (and other *Enterobacteriaceae*) biofilm formation, several cellular outer membrane (OM) structures are involved, such as fimbriae, outer membrane proteins (OMP), flagella, or lipopolysaccharide (LPS, endotoxin) (Nakao et al. 2012; O'Toole et al. 2000; Abdel‐Rhman 2019).

LPS is considered a major component of the Gram‐negative bacteria OM, providing it structural integrity, but also interacting with biotic and abiotic surfaces as well as with the components of the immune system (Li et al. 2017; Lindhout et al. 2009; Camprubí et al. 1993; Harvill et al. 2000). Studies have shown that LPS plays an essential role in the colonisation of the large intestine of mice (Nevola et al. 1985), invasion and intracellular replication (Hölzer et al. 2009) and protection of bacteria from complement killing (Murray et al. 2003, 2005; Joiner, Hammer, Brown, Cole, and Frank 1982; Joiner, Hammer, Brown, and Frank 1982; Grossman et al. 1987; Bravo et al. 2008; Krzyżewska‐Dudek et al. 2022; Pawlak et al. 2017). LPS is composed of three distinct parts referred to as the lipid A, core oligosaccharide, and the O‐antigen (the part that is most exposed to the environment). The O‐antigen of *S.* Enteritidis consists of repeating units (RUs) of polysaccharides made up of various sugar residues, exhibiting a high degree of variability (Raetz and Whitfield 2002; Samuel and Reeves 2003). The three main types of *S.* Enteritidis O‐antigens are: the low‐molecular‐weight O‐antigen LPS (LMW‐OAg LPS, composed of less than 15 RUs); the long O‐antigen LPS (L‐OAg LPS; 16–35 RUs); and the very long O‐antigen LPS (VL‐OAg LPS; > 100 RUs). There are many studies showing how variations in different LPS parts (especially in the core part) affect biofilm formation in various bacterial species (Nakao et al. 2012, 2006; Abdel‐Rhman 2019; Lee et al. 2016; Yethon et al. 2000; Laekas‐Hameder and Daigle 2024; Mireles et al. 2001; Clarke et al. 2023; Anriany et al. 2006). However, the results are often contradictory, sometimes indicating an increase and sometimes a decrease in biofilm production. Nakao et al. examined the biofilm formation properties of a series of *Escherichia coli* ( *E. coli* ) LPS mutants varying in the composition of the core region. A deep core mutant, defective in the biosynthesis of heptose, showed enhanced capacity to produce biofilm in comparison to the wild‐type strain, which produced a complete inner and outer core polysaccharide (Nakao et al. 2012). In turn, Guard‐Bouldin et al. showed that among biofilm‐positive phenotypes of *S.* Enteritidis, strains producing low or high molecular mass LPS can be identified (Guard‐Bouldin et al. 2004). Unfortunately, the methodology applied in this study, based on the detection of rhamnose, was not able to discriminate between various LPS O‐antigen forms in the tested strains, only characterising the average length of LPS O‐antigen rather than providing a precise measurement. As a result, their research did not offer a definitive answer to the critical question of how the type of O‐antigen influences *S.* Enteritidis biofilm formation. To our knowledge, so far, it has not been systematically investigated what role the O‐antigen chain length of *S.* Enteritidis might play in biofilm formation. Given the limited understanding of this potential correlation, our study aims to bridge this gap, as progress on this issue is crucial not only for advancing our scientific understanding of *S.* Enteritidis but also for the future potential in improving diagnostics of *S.* Enteritidis virulence, food safety protocols, and reducing the economic burden associated with salmonellosis. In addition to the investigation of the relationship between the O‐antigen chain length and biofilm formation properties of *S.* Enteritidis, we also investigated whether the shortening of the O‐antigen chain length would affect the OM proteome of *S.* Enteritidis.

## 2 Experimental Procedures

### 2.1 Bacterial Strains

**FIGURE 1:**
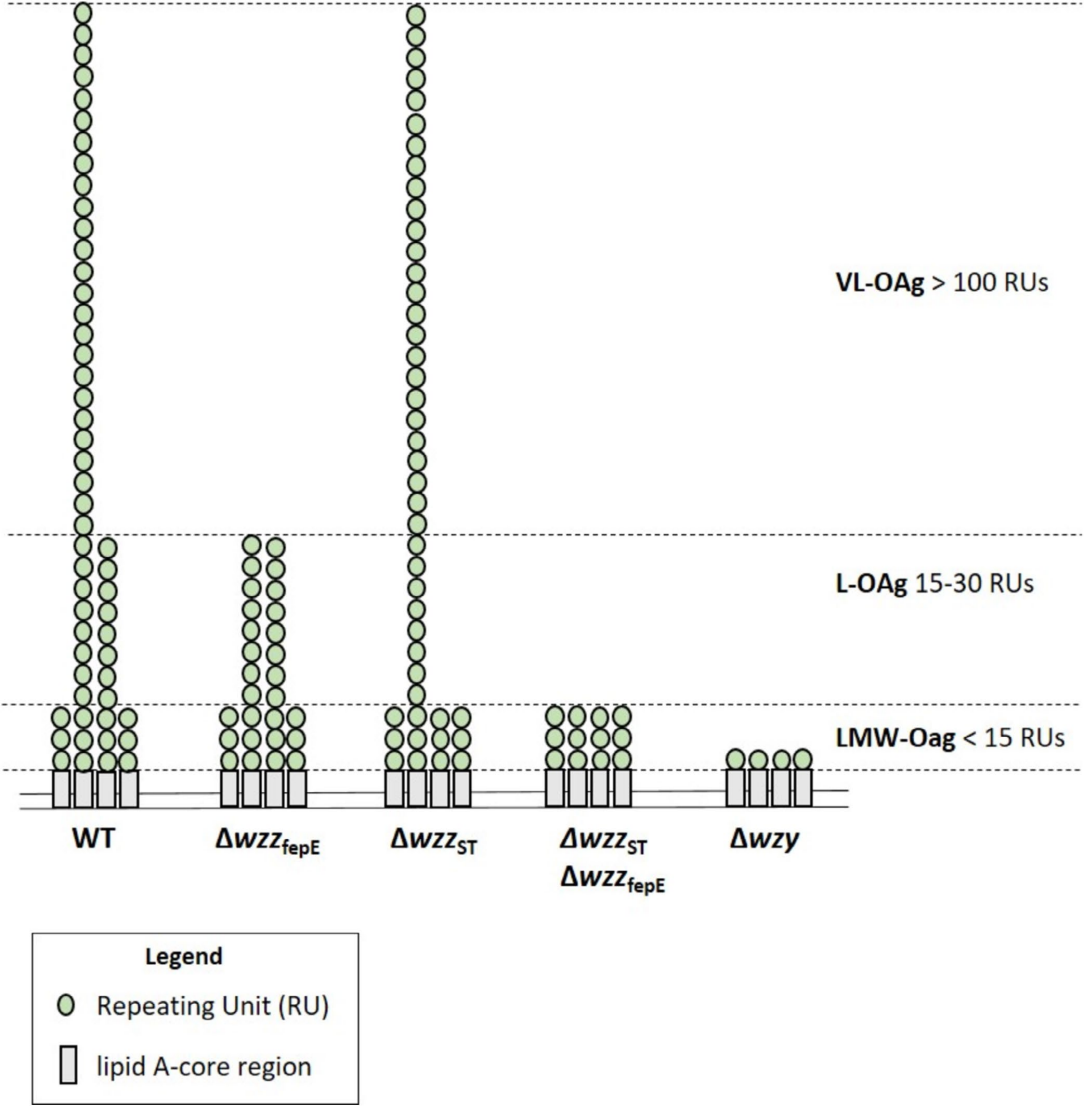
Schematic overview of the LPS O‐antigen chain length mutants of *S.* Enteritidis PCM 2817. LMW‐OAg: low molecular weight O‐antigen LPS; L‐OAg: long O‐antigen LPS; VL‐OAg: very long O‐antigen LPS; WT: wild‐type *S.* Enteritidis PCM 2817.

The panel of *S.* Enteritidis PCM 2817 O‐antigen chain length mutants characterised in detail in our previous study (Krzyżewska‐Dudek et al. 2024) was applied also in the current research. Briefly, the LPS profile of the wild‐type *S.* Enteritidis PCM 2817 strain (WT) is characterised by three separate fractions: VL‐OAg, L‐OAg, and LMW OAg LPS. The knockout of genes responsible for the chain length regulation (*wzz*_ST_, *wzz*_fepE_, *wzy*) in *S.* Enteritidis resulted in the creation of four O‐antigen chain length mutants: Δ*wzz*_ST_, Δ*wzz*_fepE_, Δ*wzz*_ST_ Δ*wzz*_fepE_, and Δ*wzy*, characterised by different LPS O‐antigen fractions. The Δ*wzz*_fepE_ mutant produced only L‐OAg and LMW‐OAg LPS, while the Δ*wzz*_ST_ mutant produced only VL‐OAg and LMW‐OAg LPS. The double knockout mutant Δ*wzz*_ST_Δ*wzz*_fepE_ produced only LMW‐OAg LPS. The O‐antigen of the Δ*wzy* mutant composes only of a single RU (Figure 1).

### 2.2 Isolation of LPS and Analysis by Polyacrylamide Gel Electrophoresis

LPS was isolated according to the method of Yi and Hackett (Yi and Hackett 2000). Briefly, 10 mg of dry bacterial mass from each strain cultured in BHI + G and LB medium was suspended in 200 μL of Tri‐Reagent (Sigma‐Aldrich, Burlington, MA, USA) and incubated at ambient temperature for 10 min. Next, 200 μL of chloroform (Chempur, Piekary Śląskie, Poland) was added, and the sample was vigorously vortexed for 10 min. The mixture was centrifuged (10 min, 14,000 × *g*) to create a phase separation. The aqueous phase was collected. Next, 100 μL of distilled water was added to the organic phase, and the sample was again vortexed for 10 min and centrifuged (10 min, 14,000 × *g*). The aqueous phases from both steps were combined and lyophilized. The samples were purified with 500 μL of cold 0.375 M magnesium chloride (POCh, Gliwice, Poland) in ethanol and centrifuged (15 min, 14,000 × *g*, 4°C). The pellet was resolved in 200 μL of distilled water and lyophilized. LPS extracts were analysed by SDS‐PAGE using the Laemmli buffer system (Laemmli 1970). Electrophoresis was performed using 6% polyacrylamide stacking gels and 15% separating gels. The SDS‐PAGE separation of LPS was performed at constant voltage (120 V) for 90 min using a Mini‐Protean Tetra Cell apparatus (Bio‐Rad, Hercules, CA, USA). The separated LPS was visualised using silver staining according to Tsai and Frasch (Tsai and Frasch 1982) with the modification of Fomsgaard (Fomsgaard et al. 1990) and imaged under white light using a GelDoc XR system (Bio‐Rad, USA).

### 2.3 Biofilm Formation Assays

#### 2.3.1 Culture Conditions

*Salmonella* strains were grown in Brain Heart Infusion (BHI) (VWR, Radnor, PA, USA) broth, supplemented with 1% glucose (Chempur, Poland) (BHI + G) or lysogeny broth (LB) (sodium chloride 5 g/L (Stanlab, Lublin, Poland), tryptone (Becton Dickinson, Franklin Lakes, NJ, USA) 10 g/L, yeast extract 5 g/L (VWR, USA)). The cultures were incubated at 37°C under static conditions.

#### 2.3.2 Assessment of Formed Biofilm Biomass Using Crystal Violet Staining

Planktonic cultures were prepared in LB and BHI + G medium. Suspensions of 0.5 McFarland density (Densitomat II, BioMerieux, Warszawa, Poland) were prepared from 24 h planktonic cultures. The suspensions were diluted to a density of 1 × 10^5^ CFU/mL in the appropriate microbiological medium. 200 μL of such bacterial suspensions were added to 8 wells of a non‐treated 96‐well flat bottom cell culture polystyrene plate (Nest, Wuxi, China). The plate was incubated for 24 h at 37°C. Non‐adhered bacterial cells were gently pulled away. Then, 200 μL of a 20% aqueous solution of crystal violet (Aqua‐med, Łódź, Poland) was added to each well and incubated for 10 min at ambient temperature. Subsequently, the non‐absorbed staining solution was removed, the wells were rinsed twice with 100 μL of 0.9% NaCl (Stanlab, Lublin, Poland), and the drying process was repeated. Next, 200 μL of a 30% aqueous acetic acid solution (Chempur, Poland) was added to each well and the plate was shaken for 30 min at 400 rpm (Schuttler MTS‐4, IKA, Staufen, Germany). 100 μL of the coloured solution was transferred to a new 96‐well plate. Absorbances were measured at a wavelength of *λ* = 550 nm (MultiScan Go, Thermo Fisher Scientific, Waltham, MA, USA). The absorbance of the 30% aqueous acetic acid solution was subtracted from the obtained absorbance values.

#### 2.3.3 Assessment of Bacterial Metabolic Activity Using 2,3,5‐Triphenyltetrazolium Chloride (TTC) Reduction Assay

The biofilm cultures were prepared in the same way as for crystal violet staining. After removal of medium, 200 μL of 0.1% tetrazolium salt solution (2,3,5‐triphenyl‐2*H*‐tetrazolium chloride, TTC) prepared in the appropriate culture medium was added to each well. The plate was incubated for 2 h at 37°C. After the incubation time, the solution was gently withdrawn. The plate was dried for 10 min at 37°C. Then, 200 μL of a mixture of acetic acid (Chempur, Poland) and ethanol (Stanlab, Poland) (1/4, v/v) was added to each well. The plate was shaken for 30 min at 400 rpm (Schuttler MTS‐4, IKA, Germany). Then, 100 μL of the solution was transferred to a new plate and the absorbance was measured at *λ* = 490 nm (MultiScan Go, Thermo Fisher Scientific, USA). The absorbance of the mixture of acetic acid and ethanol was subtracted from the obtained absorbance values. The absorbance was measured at a wavelength of *λ* = 490 nm, and the absorbance of the acetic acid/ethanol mixture was subtracted from the obtained values (Mączyńska et al. 2010; Peeters et al. 2008).

### 2.4 Characterisation of Hydrodynamic Size and Zeta Potential of Bacterial Cells Using Zetasizer Measurements

Entire bacterial cells were characterised for the hydrodynamic size distribution (nm) by dynamic light scattering (DLS) by measuring the z‐average parameter. Zeta potential (mV) was characterised by electrophoretic light scattering (ELS). Both measurements were performed using a Zetasizer Nano ZS (Malvern Panalytical, Malvern, Worcestershire, UK), with default settings and a refractive index of 1.385. *Salmonella* strains were refreshed from an overnight culture in LB medium to an OD_600_ value of 0.35 and washed three times in phosphate‐buffered saline (137 mM NaCl, 2.7 mM KCl, 8 mM Na_2_HPO_4_, and 2 mM KH_2_PO_4_ (Sigma‐Aldrich, USA), pH 7.4) (PBS). Each measurement was performed twice in three biological replicates using automatic mode at 25°C.

### 2.5 Characterisation of Bacterial Cell Surface Hydrophobicity Using a Salt Aggregation Assay

Surface hydrophobicity of bacterial cells was estimated using the salt aggregation test according to Rosenberg et al. (1980). The method is characterising the ability of bacterial strains to interact with hydrocarbons. Briefly, *Salmonella* strains were refreshed from an overnight culture in LB medium to an OD_600_ value of 0.5. In the next step, 1.2 mL of bacterial cells was centrifuged (15 min, 2000 × *g*, 4°C) and washed three times with PBS. Next, bacterial pellets were suspended in 1.2 mL of PUM buffer (K_2_HPO_4_ × 3H_2_O (Chempur, Poland) 22 g/L, KH_2_PO_4_ (Chempur, Poland) 7.26 g/L, urea (Stanlab, Poland) 1.8 g/L, MgSO_4_ × 7H_2_O (Chempur, Poland) 0.2 g/L, pH 7.1) and the absorbance at a wavelength of *λ* = 405 nm (λ1) was measured with a spectrophotometer. In the next step, 200 μL of hydrophobic p‐xylene (POCh, Gliwice, Poland) was added to each sample and incubated for 10 min with shaking. After phase separation, the absorbance of the aqueous phase was measured (*λ*2). The higher the hydrophobicity of the cells, the lower the absorbance in the water phase (cells move to the hydrophobic p‐xylene phase). Cell surface hydrophobicity was calculated based on the equation (*λ*1−*λ*2)/*λ*1 × 100%.

### 2.6 Visualisation of Auto‐Aggregation of Bacterial Cells by Fluorescent Microscopy

High auto‐aggregation of cells may indicate a strong tendency of bacteria to form biofilm. The auto‐aggregation properties of all strains were analysed by fluorescent microscopy (Opta‐Tech, Warszawa, Poland) after staining bacteria with acridine orange (Merck KGaA, Darmstadt, Germany). Briefly, bacteria were plated on LB‐agar plates and incubated overnight at 37°C. The next day, one colony of each strain was transferred to a vial using a pipette tip, resuspended in 10 μL of PBS, and mixed with 5 μL of 0.5 mg/mL acridine orange. 5 μL of such a suspension was placed on a microscope slide, covered with a cover slip, and analysed in the blue laser spectrum at 100×, 200×, and 400× magnification.

### 2.7 Isolation of OM Proteins With Chaotropic Salts and French Press

OMP were isolated according to the method of Thein et al. using chaotropic salts and a French press (Thein et al. 2010). *S.* Enteritidis PCM 2817 wild‐type strain and mutants were cultured for 18–24 h with shaking (180 rpm) at 37°C in 20 mL of LB medium. Bacteria in the early exponential stage were transferred to 200 mL of sterile LB medium and incubated at 37°C until an optical density (OD_600_) of 0.8 was reached. The cultures were centrifuged (15 min, 3000 × *g*, 4°C), and the pellets were resuspended in 12 mL of 0.1 M Tris–HCl buffer (pH 7.3) supplemented with 14 mg of DNase (Sigma‐Aldrich, Burlington, MA, USA). A French press (American Instrument Company, Haverhill, MA, USA) was then used to disintegrate the cells (two cycles at 10^8^ Pa). The bacterial suspension was centrifuged (15 min, 3000 × *g*, 4°C), and cell debris were separated from the supernatant. The supernatant was then combined with 48 mL of ice‐cold 0.1 M Na_2_CO_3_ buffer (pH 11) and incubated for 1 h on a magnetic stirrer (ES21, Kraków, Wigo) at 4°C. After incubation, the supernatant was ultracentrifuged in a Sorvall wX+ centrifuge (Thermo Scientific) (1 h, 120,000 × *g*, 4°C). The OMP‐containing pellet was resuspended in 2 mL of 0.1 M Tris–HCl buffer (pH 7.3) and centrifuged again (20 min, 85,000 × *g*, 4°C). The pellet was then resuspended three times in 500 μL of MiliQ water and centrifuged (20 min, 85,000 × *g*, 4°C). After the final centrifugation, the proteins were resuspended in 500 μL of MiliQ water and stored at −20°C until use. For each strain three independent cultures and isolations of OMP proteins were performed. The concentration of isolated proteins was determined using the commercial Pierce Coomassie Plus (Bradford) Assay Kit (Thermo Scientific) according to the manufacturer's procedure.

### 2.8 Sample Preparation for Mass Spectrometry Analysis

#### 2.8.1 Reduction, Alkylation, Digestion, and Labelling With Tandem Mass Tags (TMT)

FASP (Filter‐Aided Sample Preparation) method was used to prepare OMP‐containing samples for mass spectrometric analysis (Wiśniewski et al. 2009). Shortly, samples containing 80 μg of protein were subjected to reduction, alkylation, and digestion on centrifugal filters with a 3 kDa molecular weight cut‐off (VWR, Radnor, PA, USA). The filter was conditioned by rinsing with 500 μL of MiliQ water and 100 μL of 100 mM TEAB (triethyl ammonium bicarbonate, Thermo Scientific, Waltham, MA, USA) buffer by spinning the filter each time after applying the solution (15 min, 10,000 × *g*, 4°C). Samples were reduced with 10 mM TCEP (tris(2‐carboxyethyl) phosphine, Thermo Scientific) for 1 h at 55°C and subsequently alkylated with 17 mM iodoacetamide (Thermo Scientific) in 100 mM TEAB for 30 min at room temperature. After incubation, the samples were centrifuged (30 min, 10,000 × *g*, 4°C) and digested with trypsin solution (protein:enzyme, 40:1) in 50 mM TEAB (Promega, Poland) at 37°C for 18 h. After digestion, the peptide concentrations were determined with the Pierce Quantitative Colorimetric Peptide Assay (Thermo Scientific). Peptides were labelled with the TMTsixplex Label Reagent Set (Thermo Scientific) according to the manufacturer's instructions. The resulting samples were stored at −80°C until further processing. All reagents and solvents were suitable for liquid chromatography mass spectrometry (LC–MS) analysis.

### 2.9 Mass Spectrometry Analysis Using LC–MS/MS

The samples were separated by nanoflow liquid chromatography using the Easy nLC 1000 apparatus (Thermo Scientific) and subjected to spectrometric analysis using the LTQ Orbitrap Elite ETD system (Thermo Scientific). The following reagents and parameters were used during the chromatographic separation: nano Acclaim PepMap 100 C18 precolumn (Thermo Scientific); Acclaim PepMap column, 75 μm × 50 cm, nanoviper (Thermo Scientific); mobile phase A: MiliQ water, 0.1% formic acid (Sigma Aldrich, USA); mobile phase B: acetonitrile:MiliQ water (90:10), 0.1% formic acid (Sigma‐Aldrich, USA); gradient: 5%–55% of phase B in 150 min; mobile phase flow: 300 nL/min, 5 μL injection. Nano‐LC flow was introduced to the NanoFlex ion source equipped with a stainless steel emitter (Thermo Scientific). MS measurements were performed in positive ion mode, in a mass range of 110–2000 using data dependent acquisition (DDA) with capillary voltage set to 3 kV. HCD fragmentation of the 10 most abundant MS peaks was employed, with normalised collision energy set to 35 eV in a 1 m/z isolation window with a minimum 2+ charge state of the parent ion and dynamic exclusion after 2 acquired MS2 spectra for 30 s. The mass spectrometer was calibrated externally using LTQ Velos Positive calibration standard with resulting SD < 1 ppm.

### 2.10 Protein Identification and Quantification Using Proteome Discoverer Software

Mass spectra were processed with Proteome Discoverer 2.4. Sequest HT engine was used to extract and annotate MS/MS spectra. Database search was conducted on Uniprot *Salmonella* Typhimurium LT2 taxonomy (accessed on 2024‐02). The following static modifications were set as method parameters: TMT6plex/+229.163 Da (Any N‐Terminus), Carbamidomethyl/+57.021 Da (C), TMT6plex/+229.163 Da (K); dynamic modification: Oxidation/+15.995 Da (M). Trypsin was used as an enzyme, with 2 allowed missed cleavage sites. Protein analysis was performed using a 10 ppm precursor mass tolerance; fragment mass tolerance was set to 0.1 Da. The false discovery rate (FDR) threshold was specified as 0.01. The identified proteins were analysed for differences in their abundance ratios in individual O‐antigen mutants in relation to the WT strain *S.* Enteritidis PCM 2817 (abundance ratio greater than or equal to 1.5 for up‐regulated proteins, abundance ratio less than or equal to 0.5 for down‐regulated proteins).

### 2.11 Functional Enrichment Analysis Using David Bioinformatics Resources

Differentially expressed proteins detected in all *S.* Enteritidis PCM 2817 LPS O‐antigen mutants were subjected to gene ontology (GO) term enrichment to investigate the biological processes (BP), molecular function (MF), and cellular component (CC) using DAVID v6.8 Bioinformatics Resources (Database for Annotation, Visualisation and Integrated Discovery) (Huang et al. 2009a, 2009b). Additionally, functional annotation clustering was performed for the up‐regulated proteins. The gene list of differentially expressed proteins was analysed with default settings (count threshold: 2, EASE threshold: 0.1).

### 2.12 Swimming Motility Assay

The swimming behavior of *S.* Enteritidis PCM 2817 strains was assessed using soft agar plates (Partridge and Harshey 2023). The investigated strains were cultured overnight at 30°C in 5 mL LB medium. After overnight incubation, 50 μL of bacterial cells were transferred into 5 mL of fresh LB medium and incubated at 30°C with rotation to achieve an OD_600_ value of 0.6. Next, 6 μL of the appropriate culture was pipetted into the centre of a soft agar plate (0.3% [w/v] agar). The plates were incubated at 30°C for ~6 h. The diameters of the bacterial growth zones were measured with a ruler, and the plates were photographed. The experiment was performed in five technical replicates.

### 2.13 Statistical Analysis

Data and graphs were analysed using GraphPad Prism (version 9.2.0 for Windows, GraphPad Software, San Diego, California USA, www.graphpad.com). Data normality distribution was tested using the Shapiro–Wilk test; ANOVA test followed by Dunnett's multiple comparisons was used to evaluate the statistical significance.

## 3 Results

### 3.1 The O‐Antigen Length of LPS Is Affecting Biofilm Formation of *S*. Enteritidis 

**FIGURE 2:**
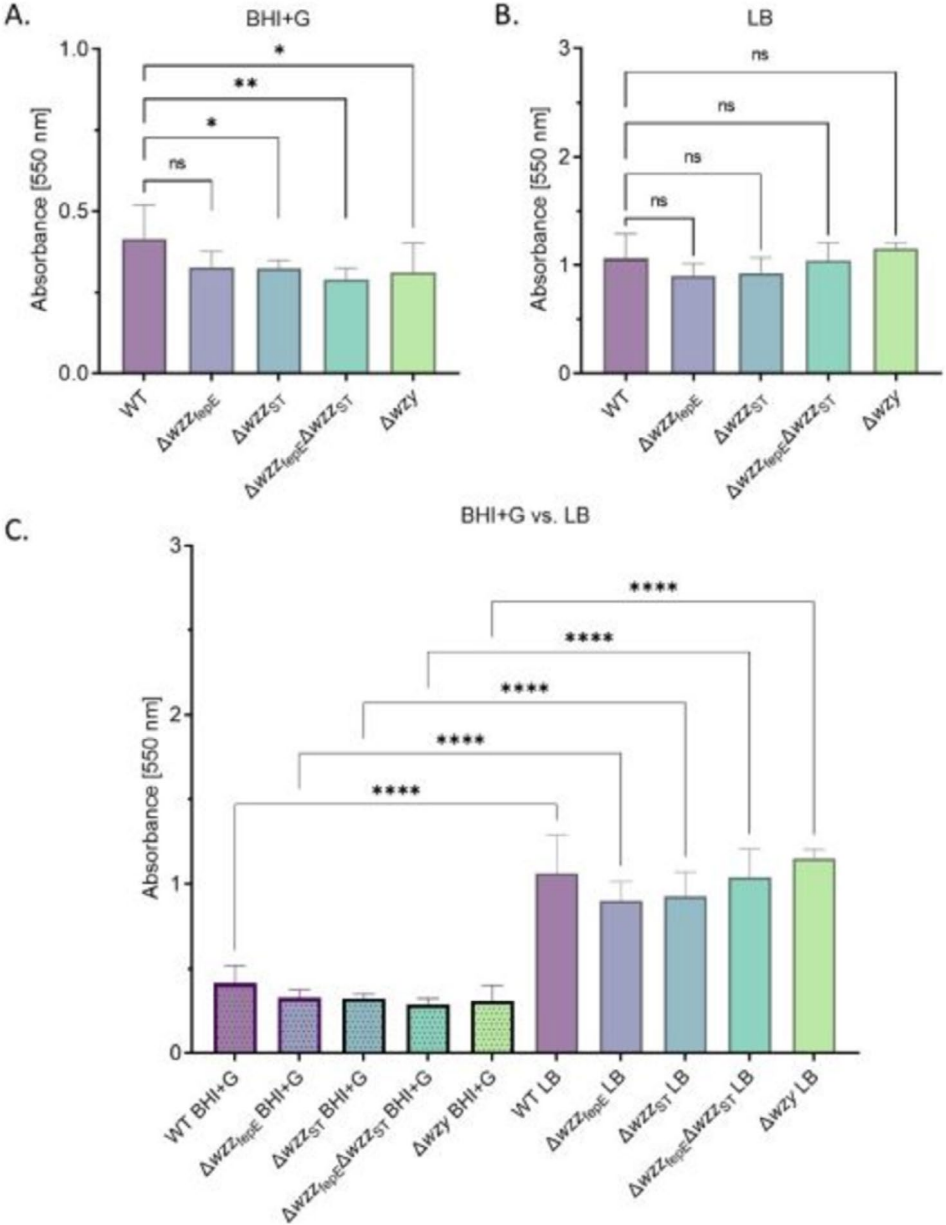
Comparison of biofilm formation properties of the tested *S.* Enteritidis O‐antigen chain length mutants assessed by crystal violet staining in two culture media. BHI + G medium (A), LB medium (B). Comparison of the biofilm formation properties in two tested media (C). Statistically significant differences are marked with asterisks (****p ≤ 0.0001).

The characteristic of the LPS O‐antigen chain lengths of the investigated *S.* Enteritidis strains is presented in Figure 1. No differences in the LPS profiles of the tested strains were observed after culture in BHI + G and LB medium (Figure S1). To investigate if the O‐antigen length of LPS affects biofilm formation, firstly, the ability of *S.* Enteritidis WT and mutant strains to bind to polystyrene in 96 h static culture conditions in both tested media was investigated and confirmed (Figure S2A). All tested strains were able to produce adherent biofilm and a slime layer (Figure S2B). Next, the biofilm biomass formed on polystyrene under stationary conditions in BHI + G and LB medium was determined using the crystal violet method (CV) (Figure 2A–C). All tested strains were able to form biofilm in a 96‐well plate in both tested conditions. The amount of biomass assessed with CV staining in the BHI + G condition was significantly lower for three out of four *S.* Enteritidis PCM 2817 O‐antigen mutants (Δ*wzz*_ST_, Δ*wzz*_ST_Δ*wzz*_fepE_, and Δ*wzy*) compared to the WT strain (Figure 2A). No statistically significant differences were observed in the level of formed biomass in the LB medium for the O‐antigen chain length mutants in comparison to the WT strain (Figure 2B). However, the obtained results indicate that the amount of biomass produced by the tested *S.* Enteritidis strains cultivated in LB medium was higher than in BHI + G medium (Figure 2C).

**FIGURE 3:**
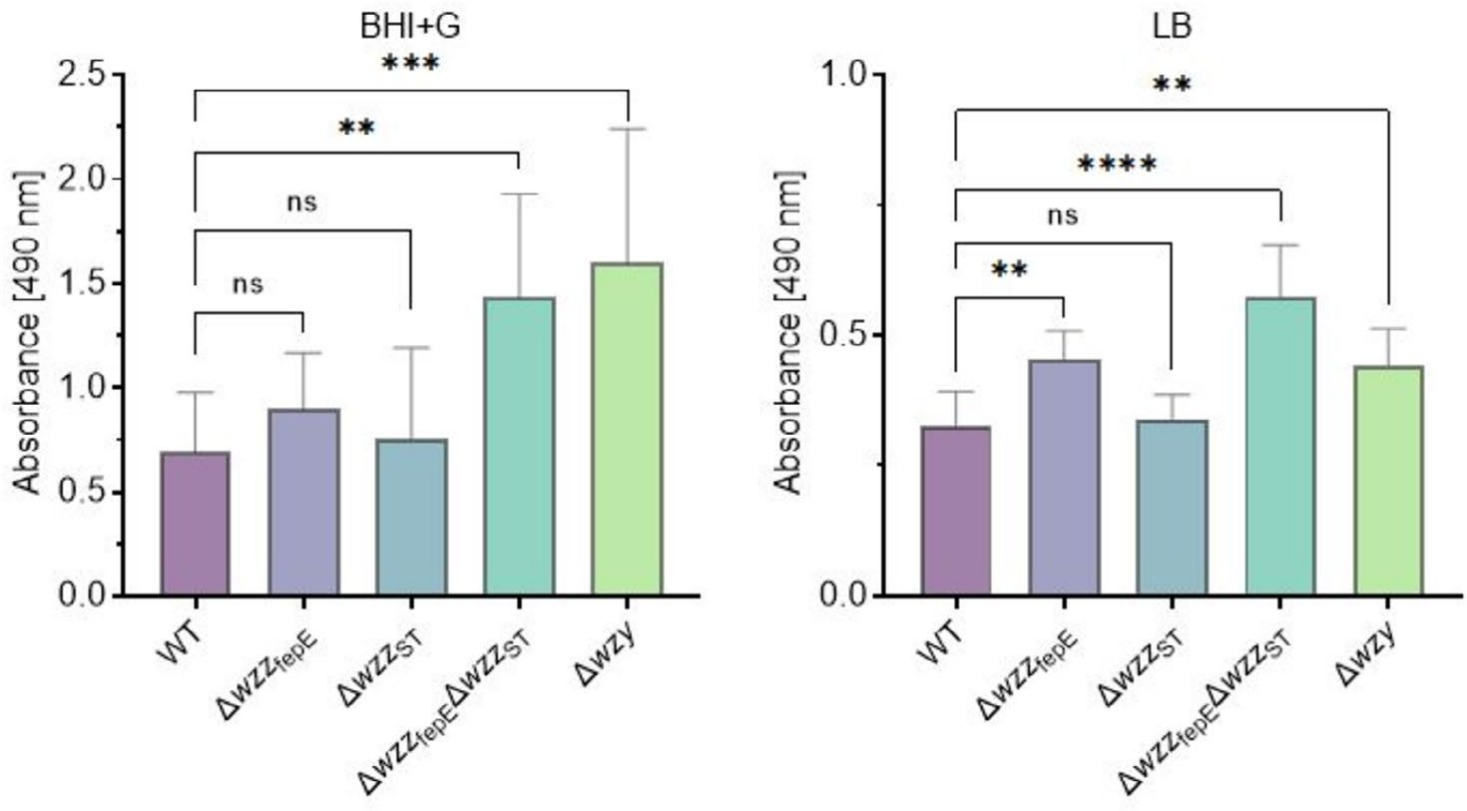
Comparison of the metabolic activity of the tested *S.* Enteritidis O‐antigen chain length mutants assessed by TCC reduction assay in two culture media. BHI + G medium (A), LB medium (B). Statistically significant differences are marked with asterisks (**p ≤ 0.01, ***p ≤ 0.001).

One of the main limitations of the usage of CV in biofilm assessment is its unspecific nature and no distinction between live and dead cells. Because of this limitation, we also assessed the metabolic activity of the tested *S.* Enteritidis strains using a TTC reduction assay (Figure 3, Figure S3). This approach allows the quantification of metabolically active bacteria and thus allows for the indirect quantification of viable cells in biofilms. The metabolic activity of biofilm formed in LB was lower than observed for biofilm cultured in BHI + G medium (Figure 3). The performed analysis showed that the tested *S.* Enteritidis O‐antigen mutants had greater variation in the results of metabolic activity. According to the results of CV staining, the double knockout mutant Δ*wzz*_ST_Δ*wzz*_fepE_ and the Δ*wzy* mutant formed lower biomass in BHI + G medium in comparison to the WT strain. However, it displayed the highest metabolic activity (Figure 3A). A higher metabolic activity could be also observed in the LB culture conditions for the Δ*wzz*_fepE_, Δ*wzz*_ST_Δ*wzz*_fepE_ and the Δ*wzy* mutant in comparison to the WT strain (Figure 3B). Interestingly, in both media Δ*wzz*_ST_ was the least metabolically active mutant. Overall, these data suggest that O‐antigen chain length is associated with biofilm formation properties in *S.* Enteritidis.

### 3.2 O‐Antigen Chain Length of LPS Influences Size, Zeta Potential, Hydrophobicity, and Auto‐Aggregation of *S. enteritidis*

**FIGURE 4:**
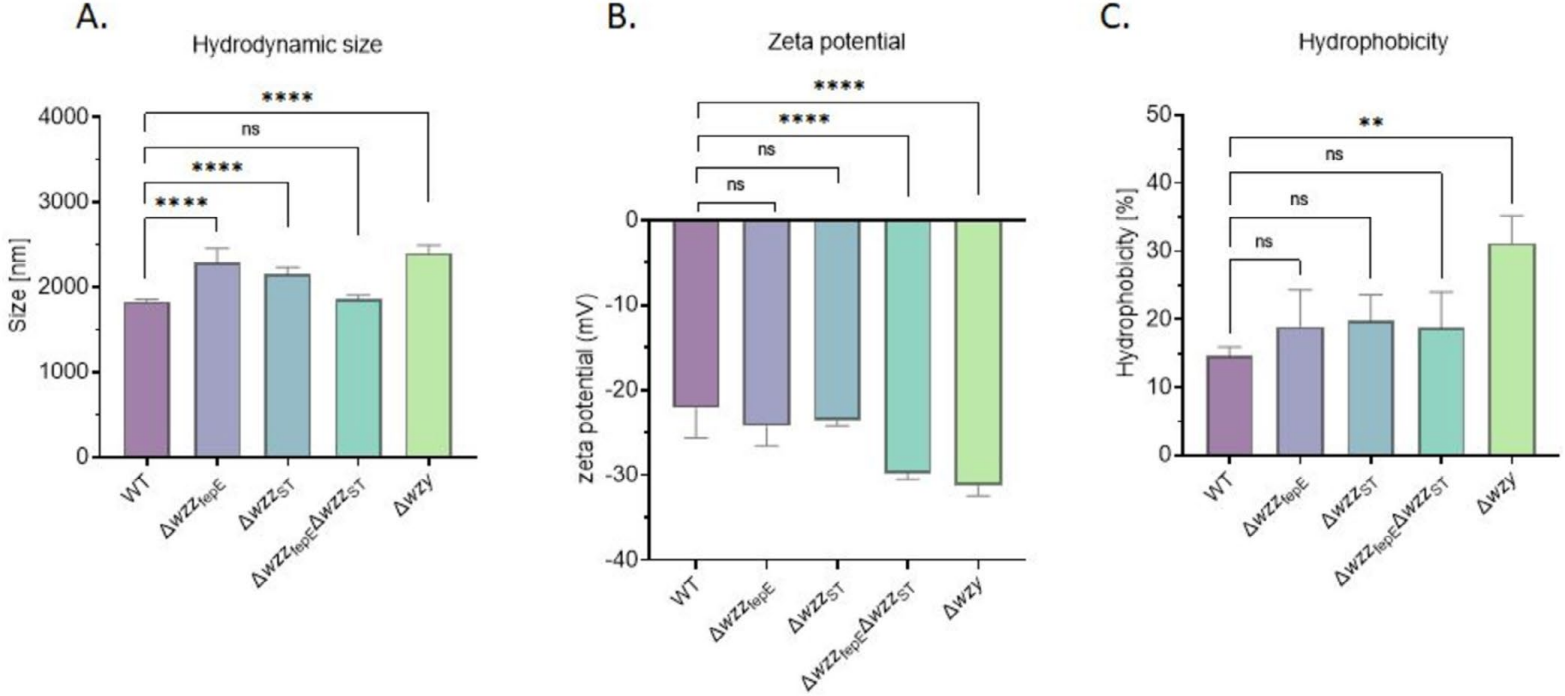
Physicochemical properties of the tested *S.* Enteritidis O‐antigen chain length mutants. (A) Hydrodynamic size, (B) zeta potential, (C) hydrophobicity. Statistically significant differences are marked with asterisks (**p ≤ 0.01, ***p ≤ 0.001, ****p ≤ 0.0001).

**FIGURE 5:**
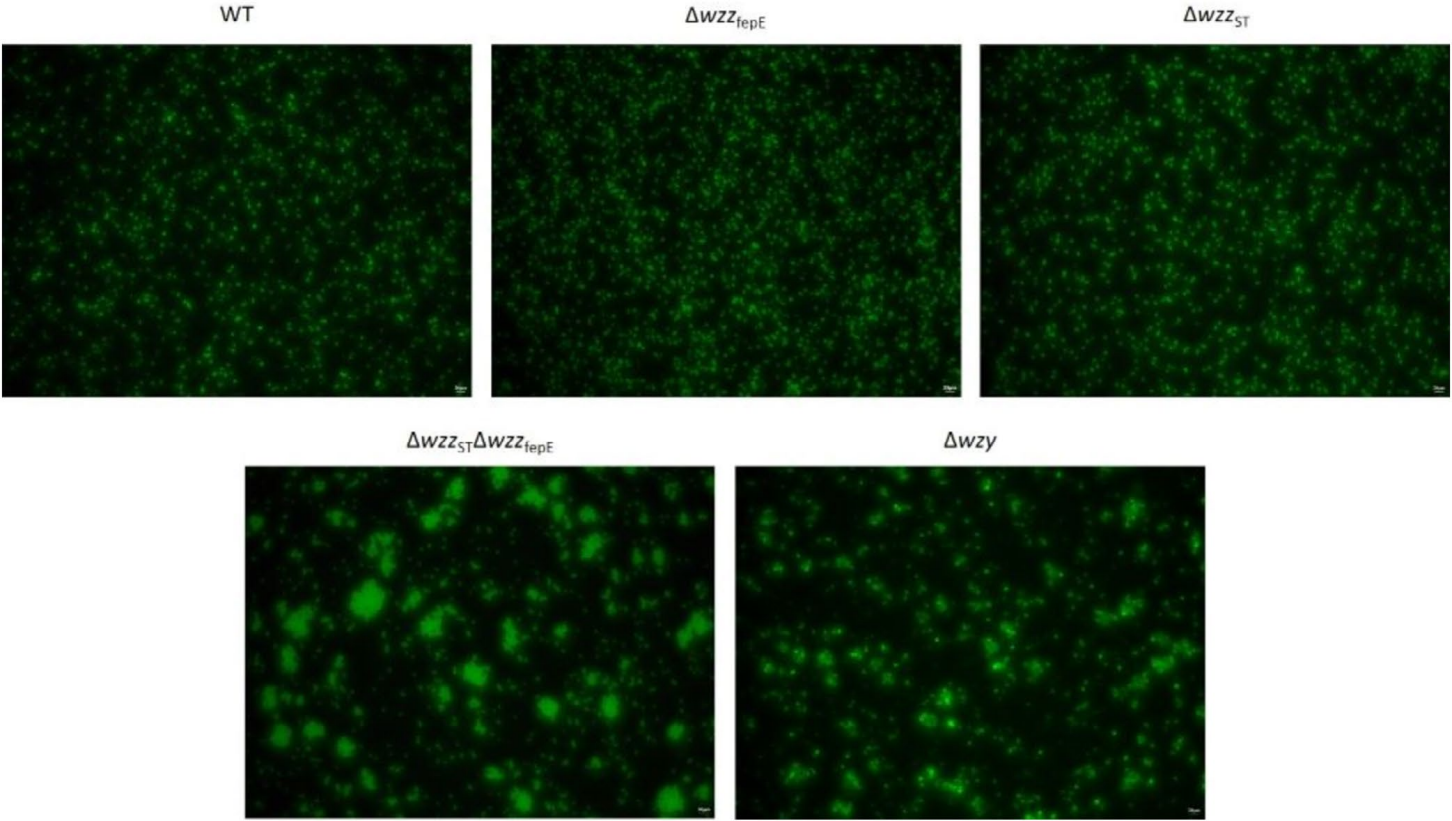
Fluorescent microscopy analysis of the auto‐aggregation properties of the tested *S.* Enteritidis O‐antigen chain length mutants. The yellow bar in lower right corner stands for 20 μm.

To get further insights into the physicochemical properties of the *S.* Enteritidis O‐antigen chain length mutants, we measured the hydrodynamic size, zeta potential, and hydrophobicity of entire bacterial cells. Hydrodynamic size and zeta potential were determined respectively by DLS and ELS. In solution, three out of four mutants with shorter O‐antigen (Δ*wzz*_fepE_, Δ*wzz*_ST_, and Δ*wzy*) were clearly larger than the WT strain (Figure 4A). Zeta potential values, which represent the electric charges at the periphery of the aggregates in suspension of the tested *S.* Enteritidis strains, varied from −22 ± 3 to −31 ± 1 mV for the WT strain and Δ*wzy* mutant respectively (Figure 4B). Overall, a decrease in zeta potential with the shortening of O‐antigen length could be detected. Hydrophobicity of entire bacterial cells was assessed by a salt aggregation test. The affinity to hydrophobic solvent was different for the tested strains. In the tested conditions, a clear trend in the increase of hydrophobicity of the O‐antigen mutants in comparison to the WT strain could be detected; however, statistical significance was detected only for the Δ*wzy* mutant (Figure 4C). We also examined the auto‐aggregation properties of the *S.* Enteritidis strains with acridine orange staining and fluorescent microscopy (Figure 5). No visible differences in auto‐aggregation could be detected for the Δ*wzz*_fepE_ and Δ*wzz*_ST_ mutants in comparison to the WT strain. In contrast, the double knockout mutant (Δ*wzz*_ST_Δ*wzz*_fepE_) and the Δ*wzy* mutant showed stronger auto‐aggregation properties than the WT strain (Figure 5). This data indicates that the substantial shortening of O‐antigen leads to an increase of auto‐aggregation in *S.* Enteritidis.

### 3.3 O‐Antigen Chain Length of LPS Is Affecting the OM Proteome Shape of *S*. Enteritidis

To get further insights into the influence of LPS O‐antigen chain length on the OM proteome of *S*. Enteritidis, we analysed the OM proteomes of the individual mutants compared to the WT strain for differences in the abundance ratios of proteins. The reference UniProt database of *Salmonella* Typhimurium LT2 used in this analysis contained 1847 reviewed records. The proteomic analysis against the reference database performed with Proteome Discoverer 2.4 (Thermo Scientific) identified 364 proteins, out of which 356 proteins had a high FDR protein confidence level (Table S1). The analysis of the abundance ratios revealed an increase in the number of differentially expressed proteins (up‐regulated and down‐regulated) with shortening of the O‐antigen chain length in comparison to the WT strain. With the assumed search parameters, 5 differential proteins were identified for the Δ*wzz*_fepE_ mutant, 10 for the Δ*wzz*_ST_, 11 for the Δ*wzz*_ST_Δ*wzz*_fepE_, and 20 proteins for the Δ*wzy* mutant. The list of differential expressed proteins is presented in Table 1. The significantly up‐regulated proteins were classified into categories using GO terms (biological process (BP), cellular component (CC), molecular function (MF)). The up‐regulated proteins were clustered into five BP terms (cellular lipid metabolic process, protein folding, cellular process, response to temperature stimulus, response to oxidative stress) and four CC terms (cell, cell part, non‐membrane‐bounded organelle, organelle). No clustering for MF terms was detected. Additionally, all up‐regulated proteins from the *S.* Enteritidis PCM 2817 O‐antigen LPS mutants were organised and condensed into functional groups. The functional annotation clustering showed five functional groups with an enrichment score greater than 0.5 (Table 2).

**TABLE 1:** Differentially expressed proteins of *S.* Enteritidis PCM 2817 LPS O‐antigen mutants.

Strain	Accession	Description	Sequence coverage [%]	MW [KDa]	Gene symbol	Abundance ratio: mutant/WT
Δ*wzz*_fepE_	P0A299	50S ribosomal protein L7/L12	36	12.3	*rplL*	2.62
	Q9ZF31	Translation initiation factor IF‐2	2	97.3	*infB*	2416
	P58480	Chaperone protein HtpG	4	71.4	*htpG*	1949
	Q8ZL46	Nucleoid occlusion factor SlmA	8	22.9	*slmA*	1814
	O30917	Chaperone protein SigE	19	12.7	*sigE*	1515
Δ*wzz*_ST_	P37416	Bifunctional ligase/repressor BirA	3	35.4	*birA*	2279
	Q9ZF31	Translation initiation factor IF‐2	2	97.3	*infB*	2.08
	P58480	Chaperone protein HtpG	4	71.4	*htpG*	1882
	Q8ZL46	Nucleoid occlusion factor SlmA	8	22.9	*slmA*	1743
	P25924	Siroheme synthase	5	50.1	*cysG*	1.71
	P52616	Phase 2 flagellin	11	52,5	*fljB*	1675
	Q8ZNF4	Lipopolysaccharide core heptose(II)‐phosphate phosphatase	3	22	*Ais*	1656
	P58663	Transcriptional regulatory protein RcsB	4	23.7	*rcsB*	1624
	P0A299	50S ribosomal protein L7/L12	36	12.3	*rplL*	1537
	Q06971	Flagellin	77	53.0	*fliC*	0.419
Δ*wzz*_ST_ Δ*wzz*_fepE_	P58480	Chaperone protein HtpG	4	71.4	*htpG*	3351
	P25924	Siroheme synthase	5	50.1	*cysG*	2359
	P16528	Transcriptional repressor IclR	5	29.7	*iclR*	2314
	P0A251	Alkyl hydroperoxide reductase C	12	20.7	*ahpC*	2253
	P0A299	50S ribosomal protein L7/L12	36	12.3	*rplL*	1978
	O30917	Chaperone protein SigE	19	12.7	*sigE*	1804
	Q8ZN53	Cytoskeleton protein RodZ	12	35.6	*rodZ*	1725
	P26982	Periplasmic serine endoprotease DegP	1	49.3	*degP*	1646
	Q8ZNF4	Lipopolysaccharide core heptose(II)‐phosphate phosphatase	3	22	*Ais*	1534
	Q8ZRP7	Vitamin B12‐binding protein	6	29.3	*btuF*	1528
	Q06971	Flagellin	77	53.0	*fliC*	0.116
Δ*wzy*	P58480	Chaperone protein HtpG	4	71.4	*htpG*	4709
	P0A299	50S ribosomal protein L7/L12	36	12.3	*rplL*	4289
	P16528	Transcriptional repressor IclR	5	29.7	*iclR*	2291
	Q8ZRP7	Vitamin B12‐binding protein	6	29.3	*btuF*	2141

	P25924	Siroheme synthase	5	50.1	*cysG*	2069
	Q8ZP52	Aconitate hydratase A	1	97.4	*acnA*	1809
	P0A210	Protein FliZ	9	21.7	*fliZ*	1731
	Q8ZMX2	Peptidyl‐lysine N‐acetyltransferase Pat	1	97.7	*pat*	1698
	Q7CPI7	Cellulose biosynthesis protein BcsG	3	62.2	*bcsG*	1658
	Q8ZNA7	Fatty acid oxidation complex subunit alpha	3	77.2	*fadJ*	1639
	Q56073	Chaperone protein DnaK	11	69.2	*dnaK*	1624
	Q8ZNF4	Lipopolysaccharide core heptose(II)‐phosphate phosphatase	3	22	*ais*	1.57
	P0A6B1	Acyl carrier protein	12	8.6	*acpP*	1561
	Q8ZN53	Cytoskeleton protein RodZ	12	35.6	*rodZ*	1.56
	O68883	Citrate synthase	7	48.1	*gltA*	1543
	D0ZV90	Virulence transcriptional regulatory protein PhoP	6	25.6	*phoP*	1543
	P58663	Transcriptional regulatory protein RcsB	4	23.7	*rcsB*	1541
	Q9L6R7	Undecaprenyl‐phosphate alpha‐N‐acetylglucosaminyl 1‐phosphate transferase	2	41.1	*wecA*	1538
	P0A1J9	Flagellar motor switch protein FliG	32	36.8	*fliG*	1532
Q06971	Flagellin	77	53.0	*fliC*	0.206

Abbreviations: kDa: kilodaltons; MW: molecular weight.

**TABLE 2:** Functional annotation clustering of differential proteins of *S.* Enteritidis PCM 2817 LPS O‐antigen mutants.

Annotation Cluster 1	Enrichment score: 1.33	Count	*p‐value*
UP_KW_CELLULAR_COMPONENT	Cilium	3	4.4E‐2
	Flagellum	3	4.4E‐2
	Cell projection	3	4.4E‐2
KEGG_PATHWAY	Flagellar assembly	3	5.3E‐2

Annotation Cluster 2	Enrichment score: 1.17	Count	*p‐value*
GOTERM_BP‐DIRECT	Protein folding	3	1.3E‐2
UP_KW_BIOLOGICAL_PROCESS	Stress response	3	4.7E‐2
GOTERM_MF_ALL	Protein binding	3	1.0E‐1
	Hydrolase activity	6	5.5E‐1

Annotation Cluster 3	Enrichment score: 0.67	Count	*p‐value*
GOTERM_CC_DIRECT	Cytoplasm	9	9.2E‐2
	Cytosol	5	5.2E‐1
GOTERM_CC_ALL	Cytoplasm	12	1.1E‐1
	Intracellular part	12	1.5E‐1
	Cytoplasmic part	12	1.5E‐1
UP_KW_CELLULAR_COMPONENT	Cytoplasm	7	2.1E‐1

Annotation Cluster 4	Enrichment score: 0.52	Count	*p‐value*
GOTERM_CC_DIRECT	Periplasmic space	3	1.2E‐1
GOTERM_CC_ALL	Periplasmic space	3	3.2E‐1
UP_KW‐DOMAIN	Signal	3	7.3E‐1

Annotation Cluster 5	Enrichment score: 0.52	Count	*p‐value*
GOTERM_MF_ALL	Nucleoside‐triphosphatase activity	4	1.2E‐1
	Organic cyclic compound binding	13	1.4E‐1
	Pyrophosphatase activity	4	1.7E‐1
	Hydrolase activity	4	1.7E‐1
	Nucleotide binding	7	2.6E‐1
	Carbohydrate derivative binding	5	4.8E‐1
	ATP‐binding	4	5.6E‐1

### 3.4 O‐Antigen Chain Length of LPS Influences the Swimming Motility of *S*. Enteritidis

**FIGURE 6:**
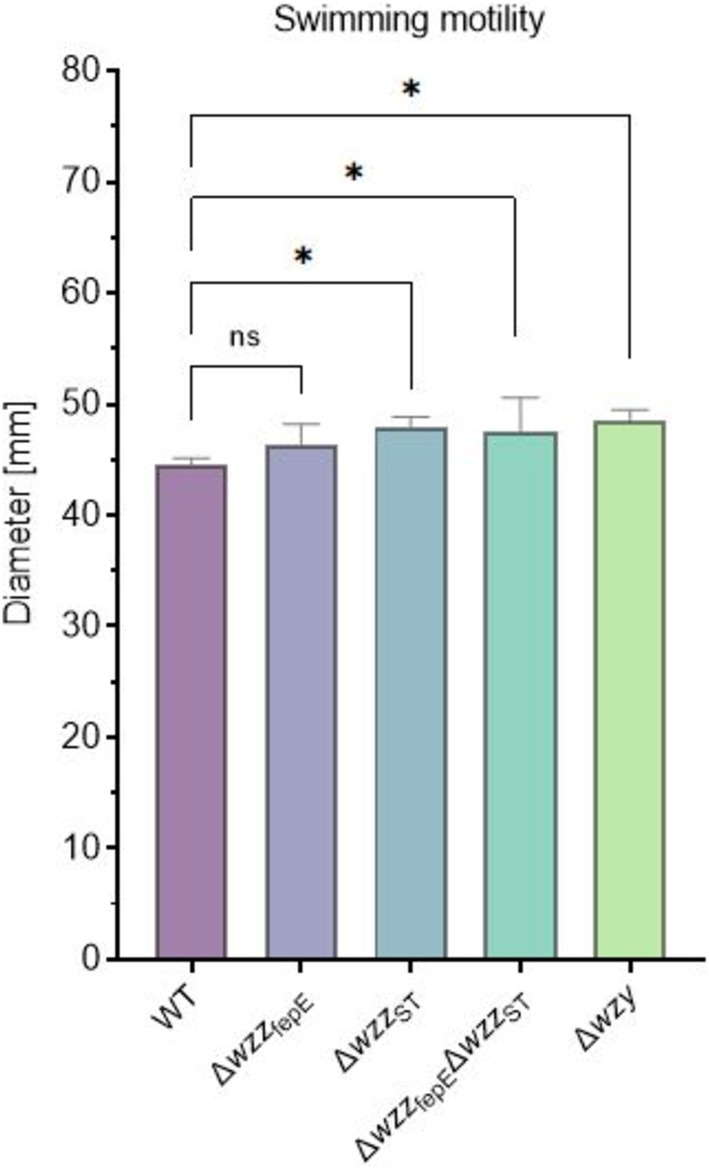
Diameters of bacterial growth zones assessed on soft agar plates of the tested *S.* Enteritidis O‐antigen chain length mutants. Statistically significant differences are marked with asterisks (*p ≤ 0.05).

The formation of biofilms is a multistep process that requires the involvement of several OM structures such as flagella and pili. To investigate if the O‐antigen chain length of LPS affects flagella‐mediated motility, we performed a swimming assay on soft agar plates. The flagellum activity of the tested *S.* Enteritidis strains ranged from 44 ± 0.5 to 49 ± 1 mm for the WT strain and Δ*wzy* mutant, respectively. Overall, a higher activity of swimming motility with the shortening of O‐antigen length could be detected (Figure 6, Figure S4).

## 4 Discussion

LPS is a major component of the cell envelope of Gram‐negative bacteria. The composition and size of the O‐antigen polymer is highly dynamic and varies between bacterial strains, playing a crucial role in the adaptation to changing environments (Trent et al. 2006; Liu et al. 2014). Here, we investigated the participation of LPS O‐antigen chain length (Figure 1) in biofilm formation and OM proteome shaping of *S*. Enteritidis.

Bacterial biofilm formation is a significant phenomenon with numerous critical implications. For instance, biofilms on the surfaces of medical devices within the body represent a serious clinical challenge, contributing to persistent infections and complications. In the context of poultry farming, biofilm formation in the farm environment serves as a source of microbial contamination, potentially leading to foodborne illnesses and the subsequent spread of disease (O'Toole et al. 2000). Bacteria in food‐processing environments may be exposed to different nutrients. The composition of the culture medium is influencing the biofilm formation properties of a given strain (Gerstel and Römling 2001; Stepanović et al. 2004). Therefore, in our study, we tested biofilm formation in a nutrient‐rich medium (BHI + G) and a nutrient‐less rich medium (LB) (Figures 2 and 3). Evaluation of the biofilm‐forming capacity of bacterial strains with different types of LPS O‐antigen chain length revealed that shortening of the O‐antigen part of the LPS molecule correlates with the decreased biofilm biomass formation in mutants with reduced O‐antigen length (Figure 2A,B) and is dependent on the composition of the culture medium (Figure 2C). The impact of medium composition on biofilm‐forming capacity and metabolic activity of various bacterial strains has been reported previously (Paleczny et al. 2022; Seneviratne et al. 2013). Paleczny et al. showed a significant reduction of biofilm biomass production and metabolic activity of *Staphylococcus aureus* ( *S. aureus* ) strains cultured in Dulbecco's Modified Eagle's Medium (DMEM) supplemented with 10% fetal bovine serum in comparison to Tryptic Soy Broth supplemented with glucose (TSB + G), pointing out the need for the implementation of adequate in vitro models that closely mimic the infection site. Additionally, the authors reported a positive correlation between the formed amount of biomass and the metabolic activity of *S. aureus* strains in the tested culture media (Paleczny et al. 2022). Our study clearly shows that the composition of the culture medium influences the amount of biofilm formed by *S.* Enteritidis. Strains grown in LB medium produced twice as much biofilm compared to strains grown in BHI + G medium (Figure 2C). However, we did not observe a positive correlation between the ability to form biomass assessed with CV and the bacterial metabolic activity measured with the TTC reduction assay in LB and BHI + G medium (Figures 2 and 3). BHI + G medium differs from LB mainly in the addition of glucose, brain and heart infusion solids, peptones, and disodium hydrogen phosphatase. Foulston et al. showed that bacterial glucose metabolism leads to increased acidity of the environment, which inhibits extracellular protease production and stimulates biofilm formation, enhancing the binding of biofilm matrix proteins to cell surfaces (Foulston et al. 2014). Interestingly, our results showed that the biofilm formation was increased in the culture medium with no added glucose (LB) (Figure 2C). A similar result was obtained by Reffuveille et al. for *S. aureus* strains, where the biofilm formation was also increased in the medium without glucose (Reffuveille et al. 2018). The discrepancy between results obtained by different researchers may be caused by diverse glucose consumption mechanisms of bacterial strains or, as reported by Waldrop et al., by a required level of glucose concentration needed for enhanced biofilm production (Waldrop et al. 2014).

In addition to the decreased biofilm biomass production with shortening of the O‐antigen part of the LPS molecule, we observed a clear increase in the metabolic activity of the tested *S.* Enteritidis strains in comparison to the WT strain (Figure 3). Interestingly, among all tested strains, the mutant with the shortest O‐antigen part (Δ*wzy*) proved to be the most hydrophobic (Figure 4C) and the most aggregative (Figure 5). Enhanced biofilm formation and auto‐aggregation by strains with truncated LPS has been shown previously for *Porphyromonas gingivalis* ( *P. gingivalis* ). Mutation in the *galF* gene resulted in a 4.5‐fold increase in biofilm formation in comparison to the wild‐type strain (Nakao et al. 2012, 2006). In contrast, the same mutation in *E. coli* resulted in higher aggregation properties but did not influence the biofilm forming and hydrophobicity of the tested strain (Nakao et al. 2012). Nakao et al. suggest that the tendency to form aggregates is connected with the enhanced cell surface hydrophobicity and that these physicochemical properties determine the extent of adhesion to abiotic surfaces such as polystyrene (Nakao et al. 2012). However, differences in the biofilm formation ability of two strains with the same mutation of the *galF* gene suggest that other effects besides hydrophobicity and auto‐aggregation may play a role in this process. Harimawan et al. suggest the surface charge is a key determinant for colonisation of a surface and biofilm forming capacity (Harimawan et al. 2011). Therefore, we measured the zeta potential of the tested *S.* Enteritidis O‐antigen chain length mutants to characterise their respective surface properties in more detail. The measurement of zeta potential gave for the WT strain (longest O‐antigen) and Δ*wzy* (shortest O‐antigen) values of −22.1 ± 3.1 and −31.2 ± 3.1 mV, respectively (Figure 4B). This result indicates that the overall negative net charge of bacterial cells with LPS containing shorter O‐antigens is greater than those of bacteria with longer LPS. It has been shown previously that shorter LPS molecules isolated from *Salmonella* Minnesota (Re595), compared to longer LPS, are characterised by a more negative zeta potential (Sali et al. 2019). Additionally, low molecular mass LPS has been described in literature by a higher hydrophobicity index and by the tendency to form larger aggregates in comparison to higher molecular mass LPS preparations (Sali et al. 2019). The obtained results, where bacterial cells with significantly shortened LPS O‐antigen (double mutant Δ*wzz*_fepE_Δ*wzz*_ST_ and Δ*wzy* mutant), having considerably decreased zeta potential (higher negative net charge of the cell surface and its higher hydration), nevertheless exhibited increased hydrophobicity, which leads to a higher number of cell aggregates in these mutants, seem counterintuitive. However, whole cells as complex systems can behave differently from small particles. The Gram‐negative bacterial membrane has a very high negative charge accumulated near the lipid bilayer (mainly from Kdo residues and phosphate groups). At the same time, the hydration of the surface is maintained by the thick polysaccharide layer of O‐antigen, even when it is built up of neutral carbohydrate residues, as in the case of *S.* Enteritidis. As the average thickness of the polysaccharide layer is decreased, the OM surface is better exposed to the environment, with both its negative charge and the hydrophobic lipids underneath. These two forces: electrostatic repulsion and hydrophobicity of the cell surface result in a decrease of zeta potential but at the same time in increased hydrophobic interactions, which leads to higher cell aggregation.

It is worth mentioning that besides the LPS structure, other cell surface molecules like flagellar and fimbrial proteins can affect zeta potential, aggregation, and biofilm‐forming properties of bacterial strains (Wyness et al. 2018; Carter et al. 2016; Jung et al. 2019). In our experiments, we have shown that the physicochemical properties of bacterial cells changed with the shortening of the LPS length in the different mutants in favour of properties promoting biofilm formation. That was confirmed by fluorescent microscopy (Figure 5), where for bacterial strains with shorter LPS molecules, the bacterial aggregates were larger and more abundant.

In this work, we also investigated whether shortening of the O‐specific part of LPS could influence the OM proteome of the bacterial cell surface and whether the up or down‐regulated proteins could influence biofilm formation. For this purpose, the OMP isolated from the tested mutants of *S.* Enteritidis PCM 2817 were subjected to a relative quantitative mass spectrometric analysis. Despite using a method for protein isolation that preferentially isolates OMP, we also identified cytoplasmic proteins in our approach (Table S1). However, a relationship could be observed between the shortening of the O‐specific part of LPS and an increase in the number of proteins with higher abundance ratios compared to the WT strain, suggesting the need to compensate for the weaker protection of the OM surface by polysaccharide chains (Table 1). The Functional Annotation Clustering of differential proteins in the O‐antigen chain length mutants revealed 5 clusters, with the highest enrichment score for a group of proteins associated with flagellar assembly (Table 2). Flagella are hairlike structures responsible for cell motility, cell surface adhesion, and biofilm formation (Subramanian and Kearns 2019; Horstmann et al. 2017; Belas 2013). Crhanova et al. demonstrated a relationship between the level of FliC protein secretion and the structure of LPS in *S.* Typhimurium bacteria. The authors showed that a deep LPS core mutant (Δ*rfaC*) was defective in FliC secretion and was characterised by a reduced number of flagellin on the bacterial cell surface, which resulted in reduced motility compared to the wild‐type strain (Crhanova et al. 2011). Additionally, the authors showed differences in the distribution of flagellin in the tested *S.* Typhimurium strains, pointing to the random distribution of the FliC protein in the Δ*rfaC* mutant along the cytoplasmic membrane and the clear concentration of the FliC protein in the wild‐type strain in spatially distinguished areas of the cell. These results indicate the influence of the LPS type on the transport of flagellin from the cell (Crhanova et al. 2011). Interestingly, in our study, FliC was the only significantly down‐regulated protein identified in 3 mutants with the shortest O‐antigen (Δ*wzz*_ST_, Δ*wzz*_ST_Δ*wzz*_fepE_, Δ*wzy*). However, other proteins associated with flagellar biosynthesis—FljB, FliZ, and FliG—could be identified with higher abundance ratios in comparison to the WT strain (Table 1). Neiger et al. compared expression levels of FljB and FliC in hyper‐biofilm isolates of *Salmonella* Typhi, showing that a low motility isolate displayed high levels of FljB and no detectable FliC. In contrast, a high‐motility isolate was characterised by high levels of FliC and low expression of FljB (Neiger et al. 2019). Western blot analysis with an anti‐*Salmonella* antibody showed the typical O‐antigen RU ladder distribution in the hyper‐biofilm isolates, suggesting the shortening or complete loss of the O‐antigen part of LPS for these strains in comparison to the wild‐type strain (Neiger et al. 2019).

Due to the fact that the Functional Annotation Clustering revealed the highest enrichment score for proteins associated with flagellar assembly, we investigated if variations in the O‐antigen length affect the motility of the tested *S*. Enteritidis strains. Our results demonstrate that shortening of the O‐antigen leads to increased motility (Figure 6, Figure S4). The obtained result partially coincides with the results obtained by Hölzer et al. where an increase in swimming motility was observed for a *S.* Typhimurium double mutant (Δ*wzz*_ST_Δ*wzz*_fepE_). Interestingly, the loss of the entire O‐antigen led to a highly reduced motility in the tested strain (Hölzer et al. 2009).

The second highest enrichment score in the Functional Annotation Clustering analysis was obtained for a group of proteins associated with protein folding, hydrolase activity, and stress response (Table 2). One of the proteins in this group was HptG. It should be mentioned that chaperon protein HptG was identified in the O‐antigen chain length mutants among the proteins with the highest abundance ratios in comparison to the WT strain. Interestingly, an increase in the abundance ratio for these proteins with the shortening of the O‐antigen could be observed, reaching a very high, regarding utilised TMT‐MS2 methodology, value of ca. 4.7 for the Δ*wzy* mutant (Table 1). HptG is a heat shock protein involved in a variety of cellular processes including stress response, signal transduction, protein folding, and repair (Grudniak et al. 2018). Dong et al. showed that the deletion of HptG in *S.* Typhimurium leads to significant reduction in biofilm formation, compromises motility, adhesion, and invasion (Dong et al. 2021). Transcriptomic analysis revealed a downregulation of genes involved in the flagellar assembly pathway (including *fliC*) after the mutation of HptG. Deletion of *hptG* resulted in reduced biofilm formation, decreased motility, and decreased activity of the LasA protease also in *Pseudomonas aeruginosa* ( *P. aeruginosa* ) (Grudniak et al. 2018). Overall, this data suggests that the up‐regulation of HptG in the tested *S.* Enteritidis O‐antigen length mutants strongly influences the biofilm‐forming capacity of the tested strains, which is accompanied by the 50S ribosomal protein L7/L12 up‐regulation, implying increased protein synthesis activity.

In some mutants, other proteins involved in LPS biosynthesis were also up‐regulated: Lipopolysaccharide core heptose (II)‐phosphate phosphatase (Δ*wzz*_ST_, Δ*wzy*) and acyl carrier protein or undecaprenyl‐phosphate alpha‐N‐acetylglucosaminyl 1‐phosphate transferase (Δ*wzy*), which suggests that the lack of certain LPS modal forms induces increased LPS synthesis to replace the lacking fractions with the shorter ones.

In our study, the Δ*wzy* mutant was characterised as the strain with the highest metabolic activity using the TTC reduction assay in both tested conditions (Figure 3) and the greatest differences in physicochemical properties compared to the WT strain (Figure 4). Among the differentially expressed proteins for this strain, aconitate hydratase A (AcnA) was identified with an abundance ratio of ca. 1.8 (Table 1). Aconitases are iron–sulphur proteins involved in the regulation of the citric acid cycle, suggesting a potential role of this protein in biofilm formation. Ball et al. demonstrated that a mutation in the *acnA* gene in *S. aureus* resulted in significantly higher biofilm formation in comparison to the wild‐type strain (Ball et al. 2022). De Becker et al. obtained results showing that the mutation of *acnA* did not lead to changes in biofilm formation under no‐flow conditions in comparison to the wild‐type strain. However, a decrease in biofilm formation was observed for this mutant under flow conditions (De Backer et al. 2018). Another protein identified in the Δ*wzy* mutant was cellulose biosynthesis protein BcsG (Table 1). Many bacteria secrete cellulose as a structural basis for biofilms (Römling and Galperin 2015). It has been shown previously that *bcsG* positively regulates biofilm formation in *Cronobacter* spp. and *S.* Typhimurium (Li et al. 2020; Sun et al. 2018). The clear up‐regulation of AcnA and BcsG in our study might indicate a contribution of these proteins to the biofilm forming capacity of *S.* Enteritidis strains with shorter O‐antigens.

In conclusion, we demonstrated that the stress induced in bacteria by the shorter O‐antigen affected the biofilm formation of *S.* Enteritidis. Additionally, we demonstrated that O‐antigen chain length may influence the composition of the OM proteome. The presented study highlights that the length of lipopolysaccharide O‐antigen is an important feature of Gram‐negative bacteria affecting their behavior and physicochemical properties of their cells.

## Author Contributions

**Eva Krzyżewska‐Dudek:** conceptualization, investigation, writing – original draft, funding acquisition, methodology, validation, visualization, writing – review and editing, formal analysis, project administration, data curation, resources. **Bartłomiej Dudek:** conceptualization, investigation, writing – original draft, methodology, validation, writing – review and editing, formal analysis. **Katarzyna Kapczyńska:** investigation, methodology, validation, writing – review and editing, formal analysis. **Paweł Pasikowski:** investigation, methodology, validation, writing – review and editing. **Malwina Brożyna:** investigation, methodology, validation, writing sss– review and editing. **Justyna Paleczny:** investigation, methodology, validation, writing – review and editing. **Agata Mikołajczyk‐Martinez:** investigation, methodology, validation, writing – review and editing. **Adam Junka:** investigation, writing – original draft, methodology, validation, writing – review and editing, supervision, formal analysis. **Jacek Rybka:** resources, supervision, writing – original draft, writing – review and editing, conceptualization, formal analysis, project administration, methodology, funding acquisition.

## Conflicts of Interest

The authors declare no conflicts of interest.

## Supporting information


**Figure S1:** LPS O‐antigen length types of *S.* Enteritidis strains cultured in LB and BHI + G medium.
**Figure S2:** A. Visualisation of the biofilm formation of the tested *S.* Enteritidis O‐antigen chain length mutants on polystyrene under static conditions. BHI + G medium (upper panel), LB medium (lower panel). B. Representative picture showing slime layer formation and adherent biofilm formation for the WT and Δ*wzy* mutant.
**Figure S3:** Visualisation of the comparison of the metabolic activity of the tested *S.* Enteritidis O‐antigen chain length mutants assessed by TTC reduction assay in BHI + G medium (upper panel) and LB medium (middle and lower panel).
**Figure S4:** Swimming motility of the tested *S.* Enteritidis O‐antigen chain length mutants on soft agar plates (three out of five technical replicates presented).

**Table S1:** List of proteins identified with a high FDR protein confidence level.

## Data Availability

The data that support the findings of this study are available on request from the corresponding author. The data are not publicly available due to privacy or ethical restrictions.
