# Supplementary material for: The Influence of Lipopolysaccharide O‐Antigen Chain Length on Biofilm Formation Capacity and Outer Membrane Proteome Shape of Salmonella Enteritidis
Source: Environ Microbiol Rep. 2025 Nov 20;17(6):e70211. doi: 10.1111/1758-2229.70211 (PMC12631911; doi:10.1111/1758-2229.70211)
Supplement: Supplementary file 1 — Figure S1: LPS O‐antigen length types of S. Enteritidis strains cultured in LB and BHI + G medium. Figure S2: A. Visualisation of the biofilm formation of the tested S. Enteritidis O‐antigen chain length mutants on polystyrene under static conditions. BHI + G medium (upper panel), LB medium (lower panel). B. Representative picture showing slime layer formation and adherent biofilm formation for the WT and Δwzy mutant. Figure S3: Visualisation of the comparison of the metabolic activity of the tested S. Enteritidis O‐antigen chain length mutants assessed by TTC reduction assay in BHI + G medium (upper panel) and LB medium (middle and lower panel). Figure S4: Swimming motility of the tested S. Enteritidis O‐antigen chain length mutants on soft agar plates (three out of five technical replicates presented). [file EMI4-17-e70211-s001.docx]

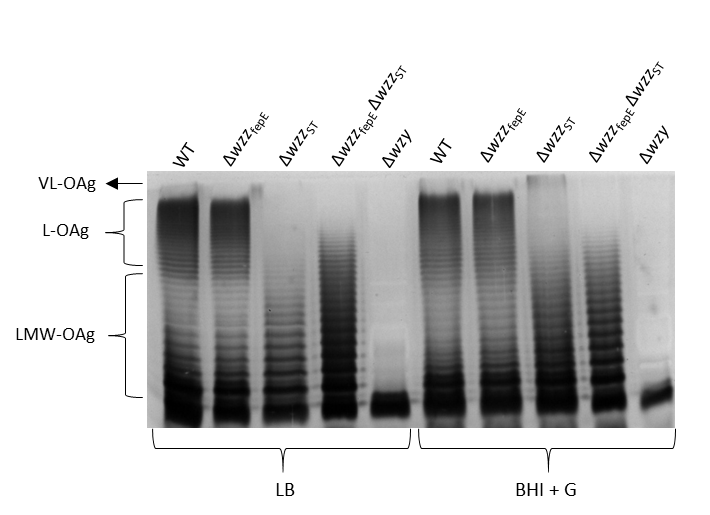


Supplementary Fig. S1. LPS O-antigen length types of *S*. Enteritidis strains cultured in LB and BHI + G medium.


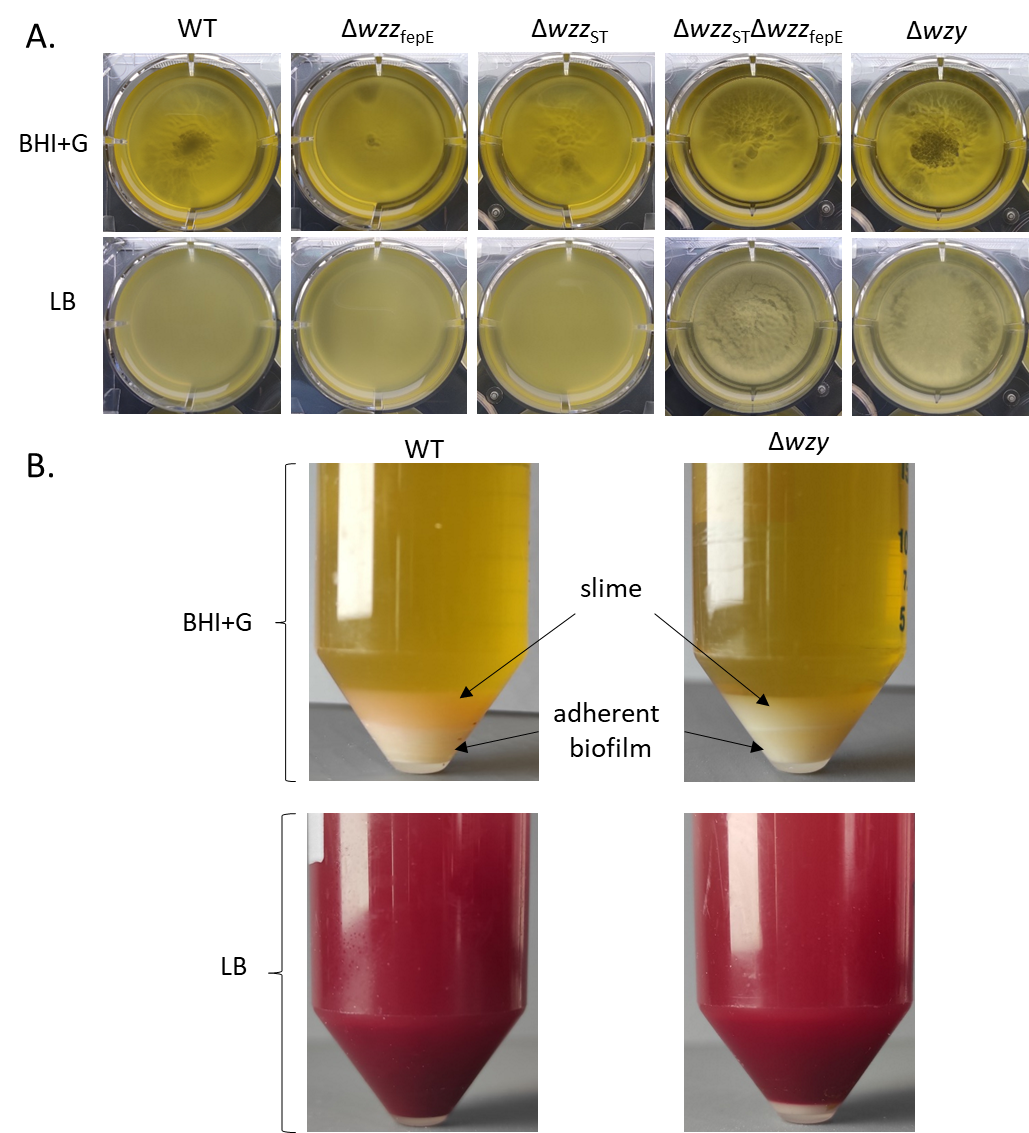


Supplementary Fig. S2. A. Visualization of the biofilm formation of the tested *S*. Enteritidis O-antigen chain length mutants on polystyrene under static conditions. BHI+G medium (upper panel), LB medium (lower panel). B. Representative picture showing slime layer formation and adherent biofilm formation for the WT and Δ*wzy* mutant .


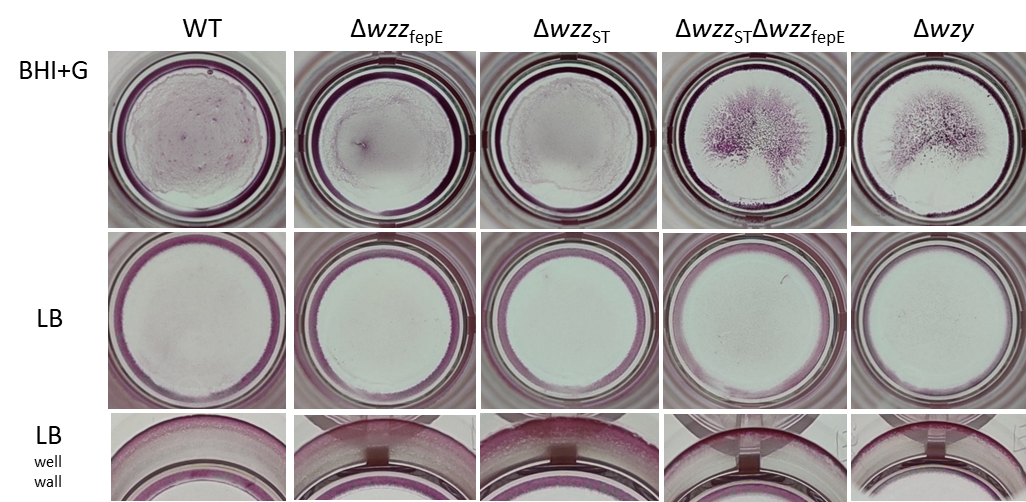


Supplementary Fig. S3. Visualization of the comparison of the metabolic activity of the tested *S*. Enteritidis O-antigen chain length mutants assessed by TTC reduction assay in BHI+G medium (upper panel) and LB medium (middle and lower panel).


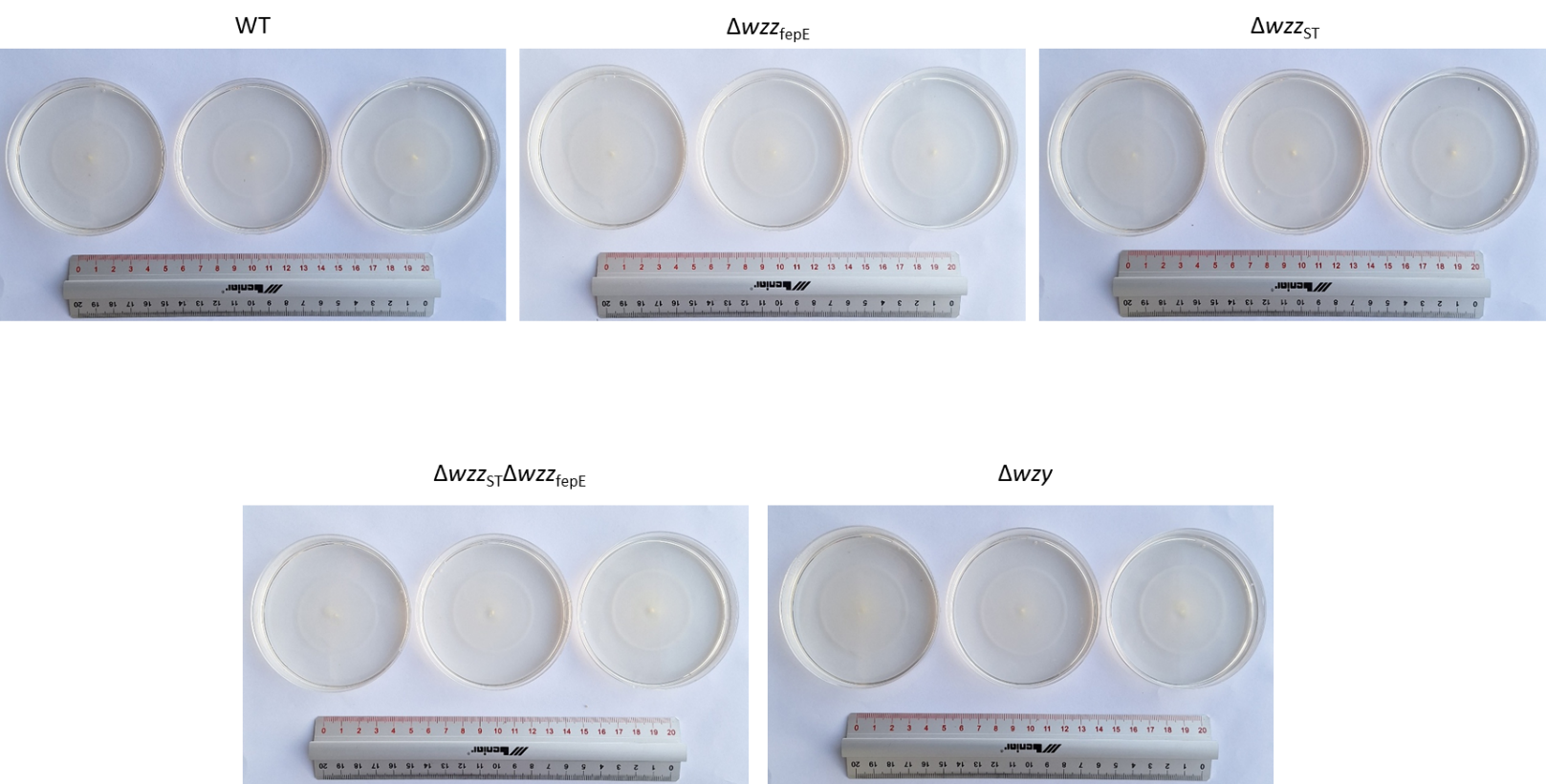


Supplementary Fig. S4. Swimming motility of tested *S*. Enteritidis O-antigen chain length mutants on soft agar plates (three out of five technical replicates presented).

Supplementary Table S1. List of proteins identified with a high FDR protein confidence level.
